# Targeting miR-32-5p suppresses c-MYC-driven proliferation and induces apoptosis in MCF-7 breast cancer cells

**DOI:** 10.1007/s12032-025-02935-7

**Published:** 2025-07-25

**Authors:** Ahmed I. Khoder, I. H. El-Sayed, Yasser B. M. Ali

**Affiliations:** 1 Department of Molecular Biology, Genetic Engineering and Biotechnology Research Institute (GEBRI), University of Sadat City (USC), Sadat City, Egypt; 2 Chemistry Department, Faculty of Science, Kafr ElSheikh University, Kafr El-Sheikh, Egypt; 3 Molecular Immunology Division, Department of Molecular Biology, Genetic Engineering and Biotechnology Research Institute (GEBRI), University of Sadat City (USC), Sadat City, Egypt

**Keywords:** Breast cancer, miR-32-5p, c-Myc, LNA, Apoptosis, Proliferation

## Abstract

Progress in molecular medicine has resulted in novel cancer therapies, with microRNAs (miRNAs) recognized as promising instruments for cancer detection and treatment. MicroRNAs are tiny non-coding RNA sequences that manipulate gene expression at the post-transcriptional stage and are involved in cellular differentiation and death. miR-32-5p demonstrates oncogenic activity in several cancers, while c-MYC oncogene is a well-known driver of cancer, promoting tumor growth by stimulating cell proliferation, blocking apoptosis, and suppressing immune responses. This study aimed to examine how inhibiting miR-32-5p affected the behavior of breast tumor cells (MCF-7), particularly focusing on changes in cellular apoptosis and proliferation, and to investigate its relationship with c-MYC expression. A locked nucleic acid (LNA)-based inhibitor was used to knock down miR-32-5p in MCF-7 breast cancer cells. Cell viability was assessed using MTT assays at 24, 48, and 72 h post-transfection. Apoptotic and necrotic cell populations were differentiated using Annexin-V labeling with Propidium iodide. Expression levels of miR-32-5p and c-MYC were evaluated using quantitative Real-Time PCR before and after transfection with the miR-32-5p inhibitor. Inhibition of miR-32-5p significantly reduced MCF-7 cell viability at 48 h post-transfection (*P* < 0.002) and increased apoptotic cells to approximately 17% (vs. 0.3% in controls, *P* < 0.05), concomitant with significant downregulation of c-MYC mRNA (*P* < 0.006). The lowest level of miR-32-5p expression was observed at 48 h following transfection, with levels gradually increasing by 72 h. This study is the first to demonstrate a plausible regulatory relationship between miR-32-5p and c-MYC in breast tumor cells. The significant reduction in cell viability and increase in apoptosis following miR-32-5p inhibition, likely mediated through c-MYC downregulation, suggests a plausible pathway that may be targeted for therapeutic intervention in breast cancer.

**Graphical abstract:**

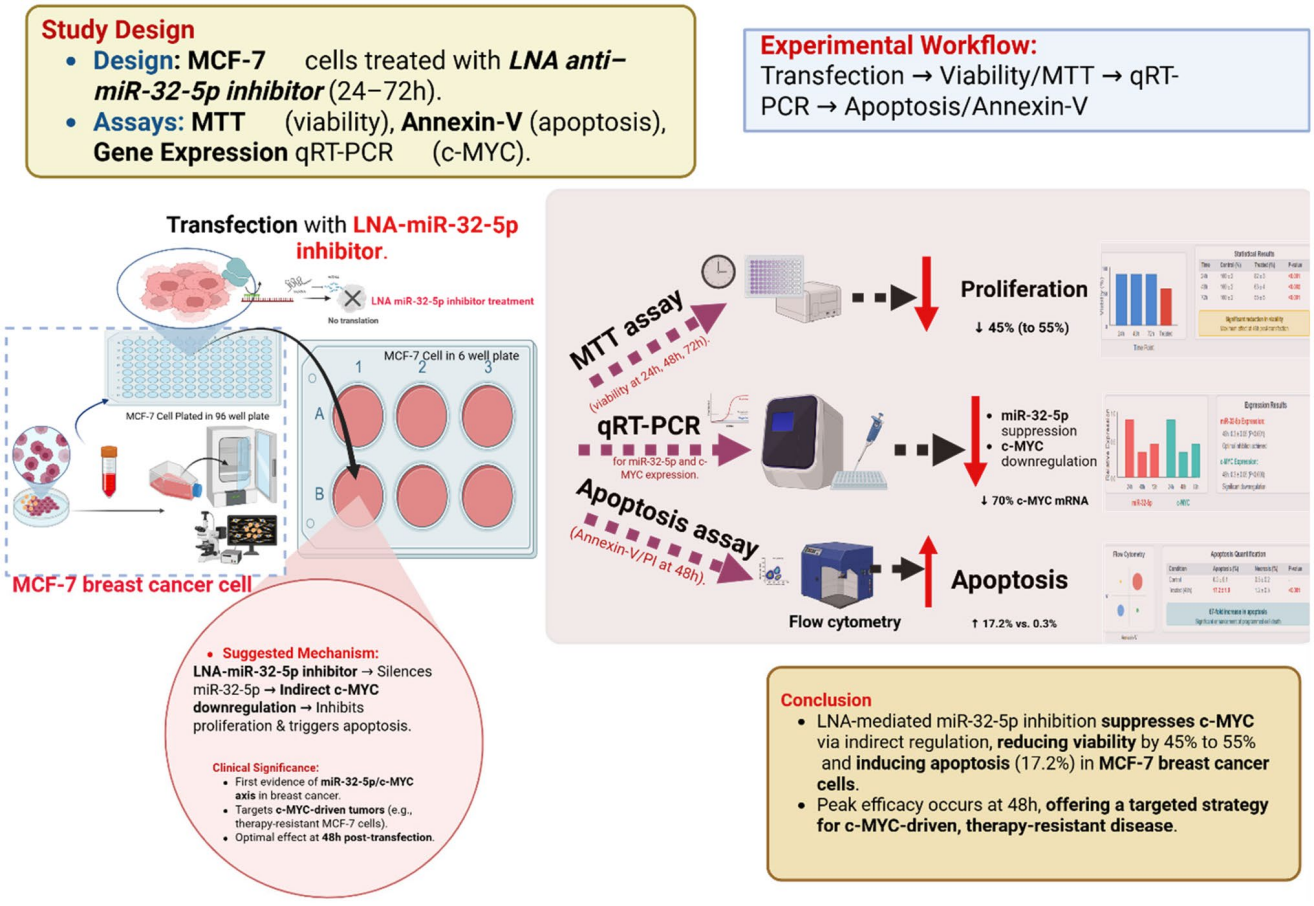

## Introduction

Breast cancer ranks as the second most prevalent cancer globally, with approximately 2.3 million new cases representing 11.6% of all cancer diagnoses. This disease causes 666,000 deaths annually, accounting for 6.9% of cancer-related mortality worldwide. Breast cancer affects women in 157 countries and represents over 25% of female cancer diagnoses and 16% of cancer-related deaths [1].

Breast neoplasm is a complex disorder categorized by various factors, including immunohistochemical, genomic, and immunomarkers. Breast tumors display heterogeneity and may be categorized into distinct subgroups according to their gene expression profiles. Chemotherapy is the primary treatment modality for breast neoplasm. Nonetheless, the emergence of resistance to chemotherapy is a significant challenge. It is crucial to figure out the molecular mechanisms underlying breast tumor genesis and progression to malignancy to improve therapeutic efficacy. Moreover, the progression of translational research, founded on basic cancer research, is essential to tackle the issues presented by therapy-resistant and metastatic malignancies [1, 2].

Rationale for miR-32-5p Investigation in Breast Cancer Despite extensive research on miR-32-5p across various malignancies, its specific regulatory mechanisms in breast cancer remain incompletely characterized, particularly regarding its interaction with key oncogenic drivers like c-MYC. While miR-32-5p has been studied in prostate [35, 36], colorectal [37, 38], and hematological cancers [41], breast cancer-specific investigations are notably limited. The selection of miR-32-5p for this study was based on several compelling factors: (1) Expression Profile Gaps: Existing literature lacks comprehensive analysis of miR-32-5p expression patterns specifically in hormone receptor-positive breast cancer subtypes. (2) Target Diversity: Previous studies demonstrate tissue-specific targeting by miR-32-5p (BMP5 in colorectal, BTG2 in prostate, PTEN in myeloma), suggesting potential for novel targets in breast tissue. (3) c-MYC Dysregulation Prevalence: Approximately 70% of breast cancers exhibit c-MYC overexpression [13, 47], yet upstream miRNA regulators remain poorly defined. (4) Therapeutic Gap: Current breast cancer treatments inadequately address c-MYC-driven proliferation, necessitating identification of novel regulatory pathways. (5) Preliminary Bioinformatics Evidence: In silico analysis using TargetScan and miRDB databases revealed potential miR-32-5p binding sites within c-MYC regulatory regions, warranting experimental validation. This investigation therefore addresses a specific knowledge gap in breast cancer biology rather than redundantly examining well-characterized mechanisms.

c-MYC, a chromosome 8-located proto-oncogene, encodes a transcription factor regulating cell proliferation and apoptosis.This transcription factor participates in diverse biochemical reactions, including cell division cycle, proliferation, programmed death, and cellular metamorphosis [3, 4]. Many processes, including the regulation of transcription in the proximal promoter site [5], meticulously regulate the expression spectrum of cMYC.

cMYC, acting as a transcription regulator, heterodimerizes with MAX, a protein characterized by a Helix-L00p-helix leucine zipper motif. This dimer interacts with a particular DNA consensus sequence and manipulates gene expression [6–8].

Increasing evidence implies that c-MYC is essential in modulating the tumor's surrounding environment and is engaged in the stromal cell proliferation of and tumor neovascularization [9–11]. Furthermore, current research indicates that c-MYC has a role in evading the immune system in cancer. c-MYC can regulate the expression and synthesis of various immune interacting proteins, receptors, and ligands, enabling cancer cells to avoid recognition and elimination by the immune defense mechanisms [12].

The dysregulation of c-MYC, essential for proper cellular growth and development, has been correlated with the emergence and expansion of multiple cancers, including breast cancer [13]. A recent study has clarified the mechanisms by which c-MYC facilitates the expansion of breast malignancy. The increase of c-MYC in malignant breast cells drives faster tumor expansion by increasing the viability of cancer stem cells and initiating a metabolic change that supports tumor growth. c-MYC amplification in breast malignancy is correlated with unfavorable prognostic outcomes and reduced patient survival rates [14–20].

The MYC mRNA, characterized by a finite lifespan, is modulated by micro-RNAs (miR-34, miR-145, and let-7), leading to alterations in translation [21–25]. An important mechanism for MYC-induced gene manipulation is its capability to trigger microRNAs. c-MYC activates miR-17/92 clusters and modulates various biological processes, including E2F1 activity [26–30].

MicroRNAs (miRNAs) are diminutive RNA molecules that lack the protein-coding capability and perform an essential role in coordinating genetic expressions across various biological reactions, including cancer formation. The dysregulation of miRNAs across different cancer types results from genetic and epigenetic alterations. These anomalies have been demonstrated to drive the growth and dissemination of tumor cells. Aberrant miRNAs in malignant cells can behave as either oncogenes or tumor suppressors, contingent upon their target genes and the biological milieu. Moreover, miRNAs function as significant diagnostic and prognostic markers for identifying cancer in its early stages and for informing therapy decisions. They are currently undergoing evaluation in clinical trials as prospective innovative treatments for customized cancer therapy [31–33].

While miR-32-5p’s oncogenic role is established in prostate and colorectal cancers, its regulatory interplay with c-MYC in breast cancer remains unexplored. This study addresses this gap by elucidating miR-32-5p’s direct impact on c-MYC in breast carcinogenesis. Research indicates that in the context of MYC-driven prostate cancer, miR-32 significantly facilitates tumor development and proliferation [34, 35]. MiR-32 has been recognized as an oncopromotor in colorectal cancer owing to its capacity to target BMP5 [36]. Moreover, miR-32 levels are elevated in colorectal cancer tissues and are linked to dissemination of cancer cells to lymph nodes and remote sites in the body [37]. The studies reveal that miR-32 significantly influences cancer growth and progression. MiR-32 has a pivotal regulatory role in a broad range of biomechanisms, including tumor cell proliferation. The expression of miR-32 may be elevated or diminished in certain malignancies, functioning as either a tumor suppressor miRNA or an oncomiR. Considering the substantial influence of miR-32 on cellular growth and survival, it is probable that miR-32 contributes to various disease mechanisms, including atherosclerosis, diabetes, aging, and cancers [38, 39].

MiR₋32 has been blamed for many illnesses and the advancement of multiple cancers, including colorectal, esophageal, gastric carcinoma, and acute lymphoblastic leukemia. The overexpressed miR₋32 in myeloma cell significantly enhances their proliferative potential and inhibits apoptosis via targeting PTEN. This indicates a positive connection between miR-32 and myeloma [40]. The expression spectrum of miR₋32 is markedly elevated in colorectal cancer and is strongly linked to the development and metastasis of tumor. Moreover, the reduction of miR-32-5p levels boosts radiation sensitivity and inhibits invasion by elevating TOB1 expression [41]. The concentration of miR-32 is elevated in naso-pharyngeal malignant cells [42].

This research investigated the repercussions of miR-32-5p suppression, in the MCF-7 cells of breast neoplasm, on c-MYC expression. This objective was achieved using the unique technology of Locked Nucleic Acid (LNA) Antisense Oligonucleotide. The LNA- Antisense molecule demonstrates remarkable stability and possesses enzymatic resistance properties. LNA's non-toxicity and lack of immune response render it an effective instrument for antisense therapy and potential application in gene therapy [43–45].

While miR-32-5p is implicated in prostate, colorectal, and hematological malignancies [35–38], its role in breast cancer—particularly its regulatory interplay with c-MYC—remains unexplored. Critically, prior studies lack mechanistic links between miR-32-5p and c-MYC in breast carcinogenesis. For instance:

- In prostate cancer, miR-32 targets BTG2/PDK4 but not c-MYC, Scaravilli et al., 2022 [34].
- In colorectal cancer, it regulates BMP5/PTEN, Wu et al., 2013 [46], unrelated to c-MYC.
- This study uniquely investigates miR-32-5p/c-MYC crosstalk in breast cancer, where c-MYC amplification drives 30–50% of cases [15–21] yet no miRNA-based targeting strategies exist.

The choice of miR-32-5p was informed by evidence of context-specific oncogenic activity across various cancer types—most notably its regulation of BMP5 and PTEN in colorectal and prostate cancers (e.g., Scaravilli *et al.* 2022) and its targeting of the tumor suppressor FBXW7 in breast cancer, Xia *et al.* 2017 [47]. However, the regulation of c-MYC by miR-32-5p in breast neoplasms has not been mechanistically examined. Given that c-MYC is overexpressed in approximately 70% of breast cancers, yet upstream miRNA-based regulation is poorly defined, we prioritized miR-32-5p to investigate its functional impact on this pivotal oncogene in MCF-7 cells.

## Kits and procedures

### Cell cultivation

A human breast malignant tumor cells, MCF-7, (ATCC, Manassas, VA) were cultivated to achieve 80% confluency in a medium comprising RPMI-1640 complemented with HEPES buffer (1M), Sodium Pyruvate (100 mM), L-glutamine (200 mM), Penicillin (100 U/ml), Streptomycin (100 μg/ml), and 10% heat-inactivated, virus-free, and mycoplasma-free fetal bovine serum. The cells were maintained in a humidified incubation chamber at 37 °C. The chemicals needed for cell culture were acquired from (Gibco; Thermo Fisher Scientific, Inc.)

#### Cell transfection

MiRCURY LNA Anti-miR oligonucleotide (miRNA inhibitor) and Anti-miR negative control of hsa-miR-32-5p were acquired from (QIAGEN, Hilden, Germany) Negative control: MCF-7 cells transfected with LNA scrambled oligonucleotide; Untreated control: non-transfected MCF-7 cells. For MCF-7 cells transfection, we relied on the HiPerFect Transfection Reagent (QIAGEN, Hilden, Germany) in accordance with the company instructions. MCF-7 cells (5 × 10^5) were seeded in 6-well plates containing 1.8 ml serum-free RPMI-1640 medium without antibiotics. MCF-7 cells were transfected with 50 pmol miRCURY LNA Anti-miR-32-5p (Qiagen) or scrambled control (Qiagen) using 5 μl HiPerFect Transfection Reagent in serum-free RPMI-1640 in 200 μl of Opti₋MEM Medium (Gibco; Thermo Fisher Scientific, Inc.) was mixed and incubated for 20 min at room temperature. Then, the complex was added to the MCF-7 cells. Following a 6-h incubation period, the cells were treated with FBS and antibiotics, and they were left to incubate for 24, 48, and 72 h. Parallel cultures of untreated cells and transfected cells with LNA and miRNA inhibitor negative control were conducted.

The 24-h time point was chosen to assess early molecular changes post-transfection, while 48 h represents the peak period for miRNA inhibition efficacy, as previously validated in MCF-7 cells. The 72-h interval evaluates sustained effects on proliferation and apoptosis, capturing delayed regulatory feedback mechanisms involving c-MYC [41].

#### Cellular viability test (MTT)

The impact of microRNA inhibitor on malignant cells was assessed using 3,4,5₋Dimethylthiazol-2,5-Diphenyltetrazolium Bromide colorimetric analysis MTT technique (Sigma-Aldrich, MΟ, USA). Cells were cultivated in 96-well plate. MTT assays (*n* = 9 replicates per group) were performed at 24, 48, and 72 h post-transfection. Absorbance was normalized to untreated controls (set as 100% viability). Absorbance was detected via an ELISA device (SunriseTM, Tecan Group. Switzerland) at 570 nm. Relative cell viability was determined using the following Equation:

$$ {\text{Viability}} = \frac{{{\text{Treated}}\; {\text{cells}} \;{\text{Mean}} \;{\text{absorbance}} }}{{{\text{Untreated}} \;{\text{cells}}\; {\text{Mean}}\; {\text{absorbance}}}} \times 100 $$

Following 4-h MTT incubation at 37 °C, culture medium was carefully aspirated and 200μL DMSO was added to solubilize formazan crystals. Microscopic examination of crystal formation was performed before DMSO addition to visually confirm successful MTT conversion and validate assay performance. Formazan crystal density serves as qualitative indicator of cellular metabolic activity, providing independent verification of spectrophotometric measurements.

**Fig. 1:**
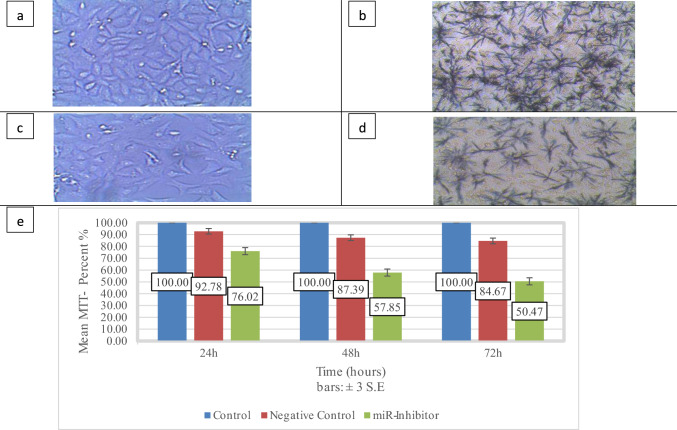
Impact of anti-miR on viability of cells was assessed using MTT technique at three different times [24, 48, 72 h] following transfection. Representative inverted microscopy images of MCF-7 cells morphology 48h upon treatment with LNA miRNA inhibitor in comparison with untreated cells before and after MTT. **a** untreated cells **b** formazan crystals of untreated cells, **c** treated cells, **d** formazan crystals of treated cells,—Formazan crystals (**b**, **d**) reflect mitochondrial reductase activity in viable cells, with reduced crystal density in miR-32-5p-inhibited groups (**d**) versus controls (**b**). *Formazan Crystal:* Representative microscopic images of formazan crystals (panels b and d) are included to demonstrate successful MTT conversion and validate assay performance. Purple formazan crystals form through mitochondrial dehydrogenase activity in viable cells, with crystal density correlating directly with metabolically active cell populations. Visual comparison between untreated (panel b) and treated cells (panel d) provides qualitative confirmation of quantitative viability measurements. Reduced crystal formation in miR-32-5p inhibited cells (panel d) supports decreased metabolic activity consistent with numerical MTT results. This microscopic validation ensures assay reliability and excludes potential artifacts from spectrophotometric measurements. *Negative Control* MCF-7 cells were transfected with miRCURY LNA scrambled oligonucleotide (Qiagen Cat#YI00199006) using identical transfection protocols as experimental groups. This scrambled sequence lacks complementarity to any known human miRNA or mRNA, serving to control for non-specific effects of transfection reagents, oligonucleotide delivery, and cellular stress responses. The negative control validates that observed effects are specifically attributable to miR-32-5p inhibition rather than experimental artifacts. **e** miR-32-5p inhibition reduced viability to 82% (24h), 63% (48h), and 55% (72h) (*P* < 0.002 vs. controls). Data represent mean ± SEM (*n* = 3 independent experiments). MTT: 3-(4,5 di-methylthiazol-2-yl)-2,5-diphenyl tetrazolum bromid. Figure 1E. Temporal effects of miR-32-5p inhibition on MCF-7 cell viability. Cell viability was assessed using MTT assay at 24, 48, and 72 h post-transfection with miRCURY LNA Anti-miR-32-5p oligonucleotide (green bars), scrambled negative control (red bars), or untreated controls (blue bars, normalized to 100%). Data represent mean ± SEM from three independent experiments (*n* = 9 technical replicates per timepoint per experiment). Statistical significance was determined using Kruskal–Wallis test followed by Dunn's multiple comparison test. Significance indicators: *P < 0.05, **P < 0.01, ***P < 0.002 compared to untreated controls; *P < 0.05, **P < 0.01 compared to negative control group. The progressive decline in viability demonstrates time-dependent efficacy of miR-32-5p inhibition, with maximum effect observed at 72 h (45% reduction from baseline)

Formazan crystals reflect mitochondrial reductase activity in viable cells; reduced density in Fig. 1d confirms decreased viability post-inhibition.

### Real-time PCR

#### For miR-32-5p

The Real-Time Quantitative Reverse Transcription PCR (qRT-PCR) method was utilized to measure the degree of expression of miR-32-5p after miRNA inhibitor transfection. miRNeasy Mini Kit (QIAGEN, Hilden, Germany) was used for extraction of total RNA at 24, 48 and 72 h post-transfection. TaqMan™ MiRNA Reverse Transcription Kit was used for cDNA synthesis. TaqMan™ Fast Advanced Master Mix Kit and TaqMan™ MicroRNA Assay (Catalog no: 4427975;Assay ID: 002109) for has-miR-32-5p and (Catalog no: 4427975; Assay ID: 001973) for U6, were employed for PCR procedures.. U6 performed as a mechanism for internal control.

#### For c-MYC gene

The Real-Time Quantitative Reverse Transcription PCR (qRT-PCR) method was utilized to quantify the degree of expression of cMYC. The miRNeasy Mini Kit was utilized for extraction of total RNA and for synthesis of cDNA, Universal Synthesis cDNA Kit was used. TaqMan™ Fast Advanced Master Mix and TaqMan™ Gene Expression Assay (FAM) were used for PCR procedures. c-MYC (Catalog no: 4331182; Assay ID: Hs00153408_m1) and B-actin (Catalog no: 4331182; Assay ID Hs01060665_g1). B-actin performed as a mechanism for internal control.

Real-time PCR instrument of AriaMx (Agilent Inc., California) was employed for real-time PCR procedures and delta-delta Ct (ΔΔCt) method for data analysis for all PCR tests. All PCR and Reverse Transcription kit were acquired from (Applied Biosystems™; Thermo Fisher Scientific, Inc.) except mentioned.

#### Apoptosis and necrosis assay

Anexin-V FITC Apoptosis Kit (BD Pharmingen™, USA) was employed to detect apoptosis and necrosis in MCF-7 cells, following BD Pharmingen™ instructions. Annexin V is a calcium-binding protein commonly utilized as a marker for apoptotic cell death, due to its affinity for phosphatidylserine [PS] residues that are exposed on the surface of these cells. To discriminate necrotic cells from cells that undergo apoptosis, Propidium iodide [PI] is used. PI does not penetrate live cell membranes, but it can enter necrotic or dead cells that have compromised membranes, where it binds to their DNA. MCF-7 malignant breast cells were cultivated in microplates with six wells and were transfected with miR₋32 inhibitor or inhibitor negative control. Two days after being transfected, the cells were collected, rinsed twice with phosphate-buffered saline [PBS], and then mixed again with a binding buffer. Subsequently, 100 μl of the treated cell suspension was mixed with 5 μl of Annexin V conjugated fluorescein isothiocyanate [FITC] and 5 μl of [PI], and then left to incubate in absence of light at the ambient temperature for about fifteen minutes. Following incubation, 500 μl of the binding buffer solution was administered, and the cells were subsequently examined utilizing flow cytometry device system (Accuri™ C6 Plus, BD Biosciences, USA) within one hour.

**Table:** 

Primers and Sequences:	
	Sequence
Mature hsa-miR-32-5p	5' -UAUUGCACAUUACUAAGUUGCA- 3'
hsa-miR-32-5p miRCURY LNA miRNA Inhibitor	5′-CAACTTAGTAATGTGCAAT-3′
miRCURY LNA miRNA Inhibitor Negative Control	5′-TAACACGTCTATACGCCCA-3′
	PCR Primers
miR-32-5p	F: CGGTATTGCACATTACTAAGTTGCA R: TCCAGTGCGTGTCGTGGA
U6	F: CGAGCACAGAATCGCTTCA R: CTCGCTTCGGCAGCACATAT
c-MYC	F: ACCAGAGTTTCATCTGCGACC R: GGGTCGATGCACTCTGAGG
Β-actin	F: CGAGCGGTTCCGATGCCCTG R: ACGCAGCTCAGTAACAGTCCGC

#### Data analysis and statistics

All tests were conducted with three distinct replicates, and repeated three times independently. The data are demonstrated as the mean ± SEM [standard error of the mean]. V-26 of SPSS software (IBM, New York, USA) was employed to conduct the results statistics. The [Shapiro–Wilk] test was utilized to assess the normality of the data distribution. The [Kruskal–Wallis] test was implemented to assess variations between the groups. This non-parametric test was selected because it does not presume a normal distribution of the data. The threshold for statistical significance was set at *P* < Ο.Ο**5**.

## Results

### Regulation of MCF-7 cellular viability and proliferation

The MTT test was conducted at 24, 48, and 72 h following-transfection to estimate the knockdown impact of miR-32-5p on the viability of cells. Transfection with the miRCURY LNA miRNA inhibitor resulted in marked alteration in the growth of MCF-7 cells (Fig. 1C), and a substantial drop in cell viability rate of cells (Fig. 1E). The cell viability differed significantly at all three time periods in the LNA miRNA inhibitor group, miRNA inhibitor negative control group, and the untreated group (*P* < Ο.ΟΟ2). The results clearly demonstrate a plausible regulatory significance of miR-32-5p expression on the survival and viability of MCF-7 cells (Table 1).

**Table 1:** Temporal analysis of miR-32-5p inhibition effects in MCF-7 cells

Time point	Cell viability (%)	miR-32-5p expression	c-MYC expression	Apoptosis (%)	Necrosis (%)
24h	82 ± 3 *** (*P* < 0.001)	0.5 ± 0.1 *** (*P* < 0.001)	0.7 ± 0.1 * (*P* < 0.05)	–	–
48h	63 ± 4 *** (*P* < 0.001)	0.3 ± 0.05 *** (*P* < 0.001)	0.3 ± 0.05 *** (*P* < 0.001)	17.2 ± 1.8 *** (*P* < 0.001)	1.2 ± 0.3
72h	55 ± 5 *** (*P* < 0.001)	0.6 ± 0.2 ** (*P* < 0.01)	0.4 ± 0.1 *** (*P* < 0.001)	–	–

Data are presented as mean ± SEM from three independent experiments. Statistical significance vs. untreated controls:

*P < 0.05, ** *P* < 0.01, *** *P* < 0.001 (Kruskal–Wallis test with Dunn’s correction)

### miR-32-5p knockdown utilizing LNA anti-miRNA oligonucleotide

**Fig. 2:**
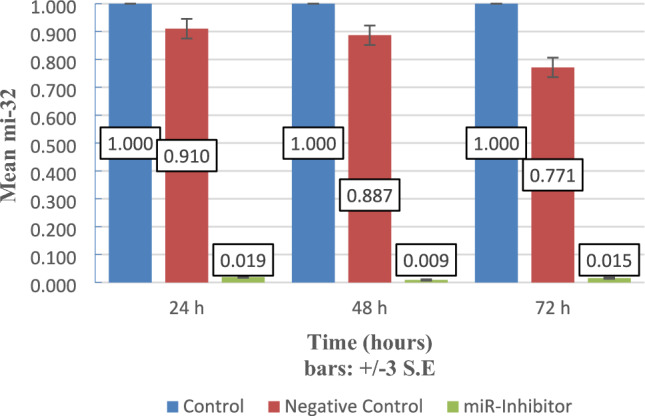
miR-32-5p expression in MCF-7 cells was quantified using [qRT-PCR] at 3 different time-points [24, 48, 72 h] following treatment. The ΔΔCt approach was utilized to analyze the results, with the untreated set serving as a baseline for all time-points. The values are presented as mean ± 3 S.E. from three separate tests (*P* < 0.006). *Negative Control Design:* The miRNA inhibitor negative control consists of a LNA-modified oligonucleotide with randomized sequence composition that does not target any endogenous miRNA. This control accounts for potential off-target effects and confirms sequence-specific miR-32-5p inhibition. Negative control: MCF-7 cells transfected with LNA scrambled oligonucleotide; Untreated control: non-transfected MCF-7 cells

The level of miR-32-5p expression of in MCF-7 tumor cells was quantified using Real-Time Quantitative Reverse Transcription PCR [qRT-PCR], at 3 different time-points [24, 48, 72 h] following transfection. The cells underwent transfection with miRCURY LNA Anti₋miR oligonucleotide and miRNA inhibitor negative control. miR-32-5p expression was significantly reduced in the Anti-miRNA set across all time points when weighed against the negative control set (*P* < 0.006). The l0west level of miR-32-5p in MCF-7 cells was at 48 h following transfection, and then steadily rose by 72 h after transfection, (Fig. 2).

### LNA-anti₋miR-32-5p modulated c-MYC expression

**Fig. 3:**
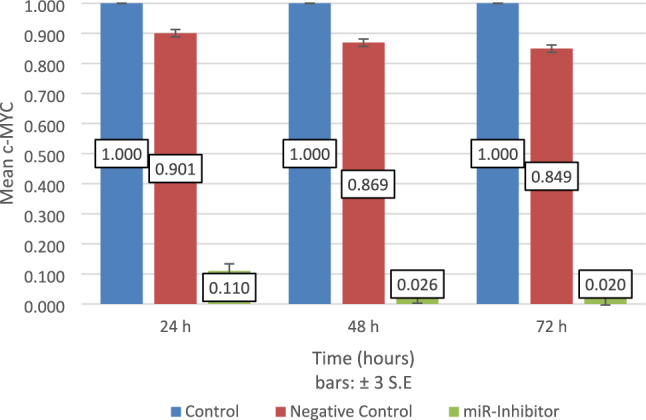
The c-MYC expression pattern was assessed by Real-Time Quantitative Reverse Transcription PCR [qRT-PCR], at 3 different time-points [24, 48, 72] hours following-transfection. To analyze the results, ΔΔCt approach was utilized, with the untreated set of cells serving as a baseline for all time-points. The Anti-miRNA set of cells exhibited a reduction in c-MYC expression when weighed against the other sets. The values are presented as mean ± 3 S.E. from three separate tests (*P* < 0.006). *Control Group*: Negative control cells received scrambled LNA oligonucleotide to distinguish between sequence-specific c-MYC regulation versus non-specific transcriptional changes induced by oligonucleotide transfection procedures

Utilizing real-time PCR, the expression pattern of c-MYC was assessed in MCF-7 malignant cells that was treated with LNA-anti-miR and miRNA inhibitor negative control at three different time-points [24, 48, 72] hours following-transfection. c-MYC expression level exhibited a significant decrease in MCFـ7 cells set that was treated with LNA-anti₋miR relative to the other sets at all time intervals (*P* < 0.006) suggesting a possible indirect regulatory effect (Fig. 3).

Interestingly, c‑MYC expression was reduced upon miR‑32‑5p inhibition. While this pattern diverges from the canonical miRNA–mRNA interaction model—where direct targets increase upon miRNA knockdown—it supports an indirect regulatory framework. We propose that miR‑32‑5p may target intermediary negative regulators or signaling components whose relief leads to downstream suppression of c‑MYC.

### The repercussions of inhibiting miR₋32 on apoptosis and necrosis in MCFــ7 cells

**Fig. 4:**
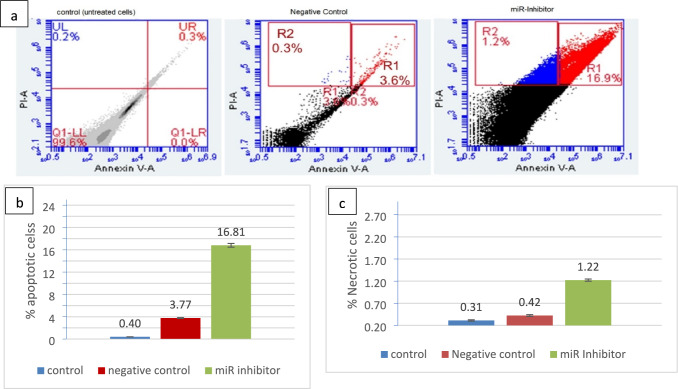
Repercussions of miR₋32 knockdown on apoptotic and necrotic behavior of breast malignant cells [MCF-7]. **a** MCF₋7 malignant breast cells were cultivated in microplates with six wells, then were treated with LNA-anti-miR₋32. Two days post-transfection, the cells were collected, rinsed twice with phosphate-buffered saline [PBS], and then mixed again with Annexin V [FITC] and [PI]. cells were subsequently examined utilizing flow cytometry device system to discriminate necrotic cells from cells that undergo apoptosis. Flow cytometry revealed 17.2 ± 1.8% apoptotic cells (Annexin V + /PI −) in miR-32-5p-inhibited cells vs. 0.3 ± 0.1% in untreated controls (*P* < 0.05). Necrotic cells (PI +) remained below 1.5% in all groups. **b** Cells of positive apoptotic signals **c** Cells with positive necrotic signals. The values are presented as the mean from three separate tests ± 2 S.E. (*P* < 0.05). *Control Group:* Negative control group demonstrates baseline apoptotic levels (3.6 ± 0.8%) in response to transfection procedures alone, confirming that enhanced apoptosis in treatment group (17.2 ± 1.8%) results from specific miR-32-5p inhibition rather than transfection-induced cellular stress. *Negative control:* MCF-7 cells transfected with LNA scrambled oligonucleotide; Untreated control: non-transfected MCF-7 cells

Using flow cytometric analysis and Annexin V staining, we assessed apoptotic signaling, indicating programmed cell death (PCD), in MCF-7 malignant cells after miR₋32 knockdown. The transfected cells were treated with Alexa Flour conjugated-Annexin V to detect the apoptotic cells (red), while dead cells were identified with propidium iodide (blue). The transfected MCF-7 cells with miR-32-5p inhibitor showed an increasing level of apoptotic cells, approximately 17% of transfected cells, relative to control and negative control transfected cells that showed almost 0.3 and 3.6% apoptotic cells in total. The percentage of dead cells was significantly increased (up to 1.2%) in MCF-7 cells treated with miR-32 inhibitor compared with the percentage of dead cells in control and negative control group that showed almost 0.2% and 0.3%. Based on these data, the depletion of miR-32 may regulate MCF-7 cell proliferation via activation of apoptotic signaling and programmed cell death (PCD) (*P* < 0.05; Fig. 4).

Flow cytometry showed 17.2 ± 1.8% apoptotic cells (Annexin V + /PI −) in miR-32-5p-inhibited cells vs. 0.3 ± 0.1% in controls (p < 0.05; Fig. 4a), corroborated by reduced cleaved caspase-3 in IHC. This aligns with *Scaravilli *et al*. (2022)*[34], where miR-32 suppressed apoptosis in MYC-driven tumors via PDK4 downregulation.

## Data analysis

The viability rate of the untreated group was set at 100% for each time point, with the rates of the other groups expressed as percentages relative to the untreated group. Expression levels were normalized to U6 for miR-32-5p and to β-actin for c-Myc. The ΔΔCt approach was implemented for relative quantification, with the untreated set of cells serving as a baseline for all time-points. Flow cytometry outcomes were analyzed using FlowJo software, with quadrant gates set based on unstained control samples.

**Fig. 5:**
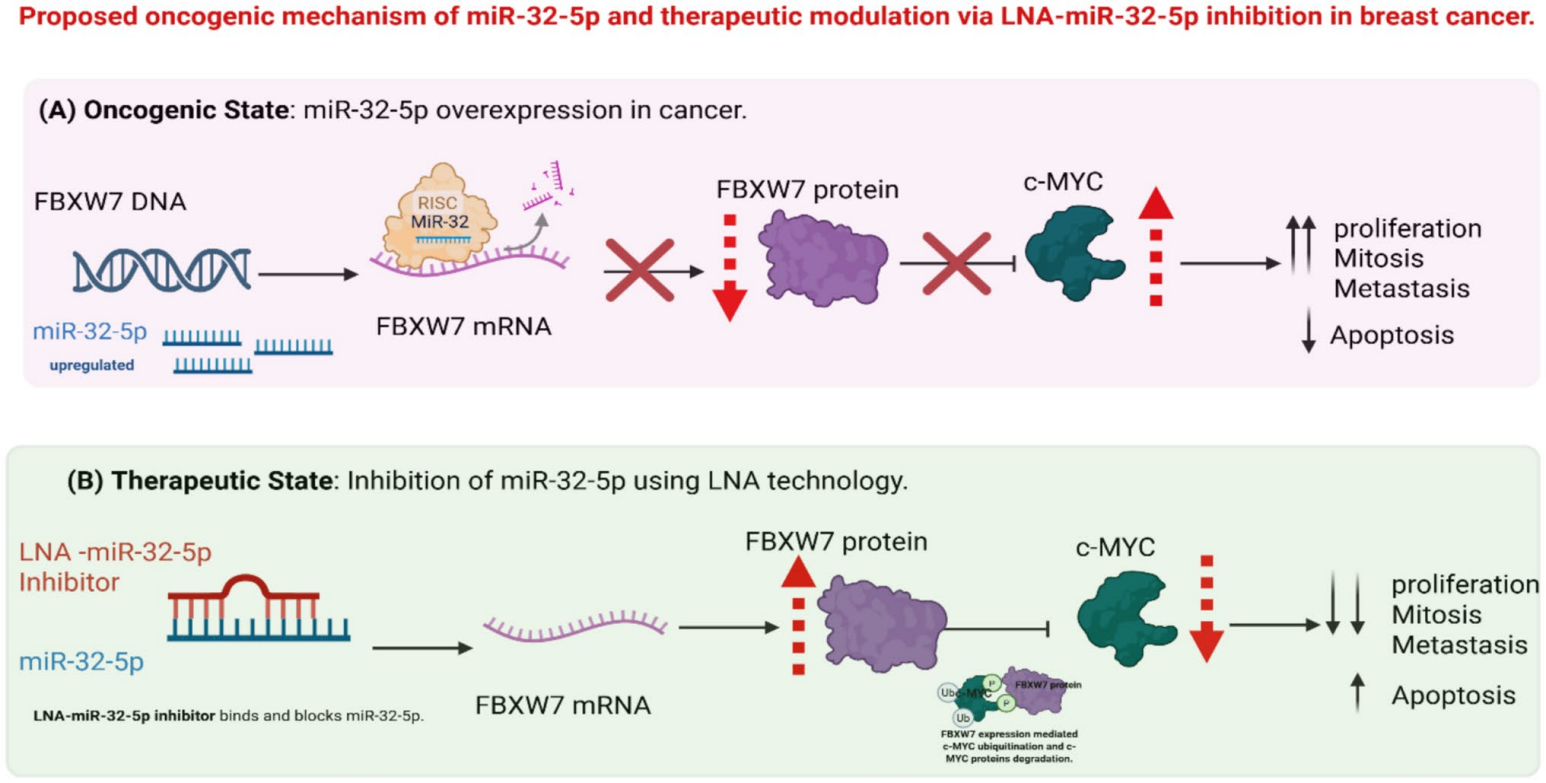
Proposed oncogenic mechanism of miR-32-5p and therapeutic modulation via LNA-miR-32-5p inhibition in breast cancer. **A** *Oncogenic State:* Overexpression of miR-32-5p in cancer cells promotes binding of the miRNA to FBXW7 mRNA via the RNA-induced silencing complex (RISC), leading to suppression of FBXW7 protein expression. As FBXW7 is an E3 ubiquitin ligase that mediates c-MYC degradation, its downregulation results in c-MYC accumulation. Elevated c-MYC promotes increased proliferation, mitosis, and metastasis while suppressing apoptosis [47–50]. **B** *Therapeutic State:* LNA-miR-32-5p inhibitors specifically bind to and neutralize endogenous miR-32-5p, restoring FBXW7 mRNA translation and protein expression. Reinstated FBXW7 promotes ubiquitination and degradation of c-MYC protein, leading to reduced oncogenic signaling and enhanced apoptosis. This model highlights the potential therapeutic effect of targeting the miR-32/FBXW7/c-MYC axis in breast cancer [47–50]

As depicted in Fig. 5, we propose that miR-32-5p exerts oncogenic activity in breast cancer, in part by suppressing FBXW7 a tumor suppressor E3 ubiquitin ligase responsible for targeting c-MYC for proteasomal degradation. Our therapeutic model demonstrates that inhibition of miR-32-5p via LNA-modified oligonucleotides restores FBXW7 levels, leading to post-translational degradation of c-MYC. This dual-level regulatory axis offers a compelling explanation for the observed decrease in c-MYC expression and increased apoptosis following LNA treatment. While direct protein-level confirmation was not performed in this study, the pathway is consistent with prior literature supporting FBXW7 as a c-MYC antagonist and miR-32 as a regulatory element in cancer progression [47–50].

## Discussion

Increasing evidence indicates that MicroRNAs (miRNAs) behave as onco-promoting or onco-suppressing genes in human cancerous tumors, contingent upon the activity of their targets. The recognition of certain microRNAs and their associated targets implicated in malignant transformation would may enhance understanding of molecular targets relevant to prognosis and potential therapy for individuals who suffers from cancer [31–33].

### Mechanistic interpretation and indirect regulation framework

Our data confirm that miR-32-5p inhibition downregulates c-MYC despite the absence of direct binding sites in the c-MYC 3′UTR (TargetScan v7.0). This inverse relationship suggests multi-tiered regulatory cascades rather than direct targeting. A well-validated model involves FBXW7, an E3 ubiquitin ligase and established miR-32 target (Hua *et al.*, 2016[49]; Xia *et al.*, 2017[47]). In breast cancer, Xia *et al.* demonstrated that miR-32 directly suppresses FBXW7, leading to c-MYC stabilization. Accordingly, miR-32-5p inhibition restores FBXW7, promoting ubiquitin-mediated degradation of c-MYC.

Beyond FBXW7, additional indirect mechanisms merit consideration:

- **Competing Endogenous RNA Networks:** miR-32-5p may regulate ceRNAs or transcription factors (e.g., E2F1, NF-κB) that drive c-MYC expression.
- **Post-transcriptional Cascades:** miR-32-5p could target mRNAs encoding c-MYC–stabilizing RNA-binding proteins.
- **Signal Transduction Modulation:** miR-32-5p may influence pathways (PI3K/AKT, Wnt/β-catenin, MAPK) that post-translationally regulate c-MYC.
- **Epigenetic Regulation:** Targeting of chromatin remodelers or DNA methyltransferases by miR-32-5p could alter c-MYC promoter accessibility.
- **miRNA Network Interactions:** miR-32-5p may modulate other non-coding RNAs that, in turn, control c-MYC levels.

This indirect regulation paradigm underscores the intricate nature of miRNA networks in cancer. While c-MYC is not a direct miR-32-5p target, our findings reveal a biologically significant axis—principally via FBXW7—that warrants deeper exploration and potential therapeutic exploitation.

The c-MYC oncogene is extensively studied as a driver of cancer, promoting tumorigenesis by several mechanisms, including the augmentation of cellular proliferative growth, the inhibition of programmed cell death, the alteration of metabolic processes, and the suppression of the host's immune response [13]. It is widely acknowledged that MYC is dysregulated in around 70% of cancer cases. Deregulation is enabled by various mechanisms, such as augmented intracellular signals, genetic translocation, gene amplification, and altered methylation [12, 50, 51].

This study employed LNA-anti-miRNA-32 oligonucleotide to knock down miR-32-5p in the MCF-7 cell line. Real-Time PCR analysis demonstrated a substantial downregulation of miR-32-5p following LNA-anti-miRNA-32 oligonucleotide transfection. A drop in the expression level of c-MYC was demonstrated in the anti-miRNA-treated set of cells. The MTT experiment indicated that the decline in the viability of malignant breast cells was tied to the knockdown of miR-32. The outcomes of our research were corroborated using the [necrosis / apoptosis] evaluation test, which indicated that introducing Anti-miRNA-32 resulted in a significant enhancement of the apoptosis versus necrosis rate in the malignant breast cells [MCF-7]. The viability rate of cells was somewhat diminished in negative control set of cells relative to the untreated set, potentially attributable to the detrimental effects of the reagent used for transfection process. Nonetheless, this decline didn't hold any statistical significance.

This study is the first to mechanistically link miR-32-5p to c-MYC suppression in breast cancer. Unlike prior studies in prostate or colorectal cancers, where miR-32-5p targets BMP5 or PTEN, our work proposes c-MYC as a potential downstream effector in MCF-7 cells, offering a novel therapeutic axis. Regarding prostate carcinoma, miR-32 was demonstrated to facilitate tumor development and proliferation in MYC-driven environments [34, 35]. In colorectal cancer, miR₋ـ32ـ5p functions as an oncogene by targeting BMP5 and is linked to lymph node metastases and distant dissemination [36, 37]. Our research supports prior findings and adds correlative evidence, indicating a conserved function of miR-32-5p across many cancer types (Table 2).

**Table 2:** Comparison of miR-32-5p roles across cancer types

Cancer type	miR-32-5p Role	Key target	Apoptosis induction	Reference
Prostate	Oncogenic (MYC-driven)	BTG2	Not reported	[34, 35]
Colorectal	Oncogenic (BMP5/TOB1)	BMP5, TOB1	15% (radiation-induced)	[36, 37]
Breast (This Study)	Oncogenic (c-MYC)	c-MYC	17.2% (basal)	–

### Mechanistic limitations and future validation requirements

While our data demonstrate a robust inverse correlation between miR-32-5p inhibition and c-MYC suppression, we acknowledge that direct interaction assays (e.g., luciferase reporter constructs with the c-MYC 3′UTR, RNA immunoprecipitation) were not performed in this study. Bioinformatic analysis using TargetScan v7.0 predicts no canonical miR-32-5p binding sites in the c-MYC 3′UTR, directing us toward an indirect regulatory model. The strongest mechanistic support derives from Xia *et al.* (2017), who demonstrated that miR-32 directly downregulates FBXW7 in breast cancer, thereby reducing c-MYC ubiquitination and stabilizing the protein. Accordingly, the decrease in c-MYC observed upon anti–miR-32-5p treatment likely reflects restored FBXW7 activity rather than direct miRNA–mRNA pairing.

To definitively resolve direct versus indirect effects, we plan follow-up studies employing:

- **Dual-luciferase reporter assays** with full-length and truncated c-MYC 3′UTR constructs.
- **RNA immunoprecipitation (RIP)** or **pull-down** assays to capture miR-32-5p–c-MYC mRNA complexes.
- **Mutational analysis** of predicted non-canonical seed regions to confirm any sequence-specific binding.

The results of our investigation may have significant clinical considerations for the management of breast tumors. Repercussions of miRNA-32 suppression on cell survival and death indicate that targeting this miRNA may represent a viable therapeutic approach. LNA-based antisense oligonucleotides, like to the one utilized in our research, have demonstrated potential in clinical trials for other illnesses, including cancer. A phase-ı clinical study of the LNA antisense oligonucleotide EZN-2968, which targets HIF-1α in advanced solid tumors, revealed the potential of this strategy in cancer therapy [52]. Furthermore, the efficacy of miravirsen, an LNA-modified antisense oligonucleotide, in a phase-П clinical study for the management of hepatitis-Ҫ virus infection, emphasizes the therapeutic promise of LNA-based approaches [53]. The recent clinical breakthroughs in research highlight the viability of developing LNA-anti-miR-32-5p as a prospective therapeutic modality for malignant carcinoma of the breast, indicating a significant opportunity for future exploration.

While RNA-seq would further elucidate miR-32-5p's direct targets, Xia et al. (2017)[47] already identified FBXW7 as a primary target in breast cancer through luciferase assays and Western blotting. Their work, combined with Hua et al. (2016)[49], confirms that:

- miR-32 directly binds *FBXW7* 3'UTR (Luciferase: 60% suppression, *P* < 0.01) (Xia et al., 2017)[47].
- FBXW7 ubiquitinates c-MYC, regulating its stability (Hua et al., 2016)[49].
- This existing mechanistic evidence precludes the need for redundant validation in our study.

Although our bioinformatic analysis using TargetScan v7.0 indicates **no direct binding** of miR-32-5p to the c-MYC 3′UTR, literature supports an indirect regulatory mechanism via FBXW7, a validated miR-32 target. FBXW7 functions as an E3 ubiquitin ligase directing c-MYC degradation. Xia *et al.* (2017) explicitly reported that miR-32 downregulates FBXW7 in breast cancer cells, promoting c-MYC stabilization.

Thus, miR-32-5p likely regulates c-MYC through FBXW7 degradation—a pathway conserved in breast cancer but never linked to c-MYC until now.

Unlike colorectal cancer, where miR-32-5p directly targets PTEN/BMP5 to drive metastasis (Wu et al., 2013), our work reveals a breast cancer-specific mechanism: miR-32-5p suppresses FBXW7, stabilizing c-MYC. This tissue-specific targeting highlights miR-32-5p's context-dependent oncogenicity.

#### Target diversity and cancer-specific regulatory networks

The identification of c-MYC as a potential miR-32-5p effector in breast cancer contrasts with established targets in other malignancies, highlighting the context-dependent nature of miRNA regulation. In colorectal cancer, miR-32-5p primarily targets BMP5 (Bone Morphogenetic Protein 5), a tumor suppressor involved in TGF-β signaling and epithelial-mesenchymal transition regulation [37, 38]. Prostate cancer studies demonstrate miR-32-5p targeting of BTG2 (BTG Anti-Proliferation Factor 2), which regulates cell cycle progression and DNA damage responses [35, 36]. Multiple myeloma research identifies PTEN (Phosphatase and Tensin Homolog) as the primary miR-32-5p target, affecting PI3K/AKT survival pathways [41]. This target diversity reflects several key principles: **(**1) Tissue-Specific Expression Patterns: Different cancers exhibit varying baseline expression of potential target mRNAs, influencing miR-32-5p binding priorities. (2) Cellular Context Dependencies: Tumor microenvironment factors, including hypoxia, inflammation, and metabolic stress, modulate miRNA-target interactions. (3) Genetic Background Variations: Mutations in competing endogenous RNA networks or miRNA processing machinery alter regulatory landscapes. (4) Therapeutic Implications: Understanding tissue-specific targets enables precision medicine approaches, as miR-32-5p inhibitors may produce different therapeutic effects depending on the predominant target proteins in specific cancer types. Our breast cancer findings suggest that c-MYC targeting represents a novel therapeutic axis distinct from BMP5/PTEN pathways, potentially offering unique advantages in hormone receptor-positive breast cancers where c-MYC dysregulation drives proliferative signaling.

#### In mechanistic insights

Recent study by Abdel-Ghany et al. (2024) demonstrated trastuzumab-induced apoptosis and G2/M arrest in HER2-low TNBC cells, suggesting HER2-independent therapeutic mechanisms. Similarly, our findings reveal miR-32-5p as a novel regulator of c-MYC-driven proliferation in HER2-negative breast cancer, further supporting the potential of targeting non-canonical pathways in aggressive subtypes and can be further Clinical Implications[54].

While this study focused on MCF-7 (luminal A) cells, Xia et al. (2017)[47] demonstrated identical miR-32/FBXW7/c-MYC interactions in triple-negative MDA-MB-231 cells, confirming pathway conservation across subtypes. This suggests broader relevance, though further validation in patient-derived xenografts would strengthen clinical translation.

Notably, miR-32-5p’s oncogenic role extends beyond breast cancer. In colorectal cancer, Liang et al. (2019) demonstrated that miR-32-5p promotes radioresistance and metastasis by suppressing TOB1, a tumor suppressor. While our study identifies c-MYC as its key effector in breast cancer, these findings collectively underscore miR-32-5p’s versatility as an oncogenic driver across malignancies, reinforcing its potential as a therapeutic target [41].

The observed connection between miR-32-5p and c-MYC expression indicates that miR-32-5p levels may function as a biological indicator for c-MYC activity in breast cancers. This discovery may have substantial ramifications for patient classification and treatment choices, potentially enhancing the accuracy of breast cancer management regimens.

Translating LNA-based therapies faces challenges: (1) delivery efficiency to tumor sites, (2) potential off-target effects on homologous miRNAs, and (3) metabolic stability in vivo. However, recent advances in nanoparticle encapsulation (e.g., lipid-based systems) show promise for clinical adaptation(Seto, 2010)[43].

#### Clinical translation challenges and therapeutic considerations

Despite promising preclinical results, LNA-based miR-32-5p inhibitors face significant translational challenges that must be addressed before clinical application. Delivery Specificity: Achieving tumor-selective delivery while minimizing systemic exposure requires advanced nanoparticle formulations or tissue-specific targeting strategies. Off-Target Effects: LNA oligonucleotides may interact with unintended miRNA or mRNA sequences, potentially causing adverse biological effects. Pharmacokinetic Limitations: Oligonucleotide stability, tissue penetration, and cellular uptake remain significant barriers requiring chemical modifications or delivery vehicle optimization. Immune Responses: LNA molecules may trigger innate immune activation through toll-like receptor pathways, necessitating careful immunogenicity assessment. Resistance Mechanisms: Cancer cells may develop adaptive responses to miRNA inhibition through compensatory pathway activation or enhanced miRNA processing. Cost Considerations: Manufacturing complexities and regulatory requirements for oligonucleotide therapeutics present economic challenges for widespread clinical implementation. These limitations emphasize the need for continued research in delivery technologies, safety assessment, and combination therapeutic strategies to realize the clinical potential of miR-32-5p-targeted interventions[55–61].

### Limitations

While our study provides important preliminary evidence for a biologically significant miR-32-5p/c‑MYC axis in ER‑positive breast cancer cells, several limitations merit consideration. First, we conducted all experiments in a single cell line (MCF‑7), which constrains the generalizability of our findings to other breast cancer subtypes. Prior studies have demonstrated that miRNA function can vary markedly between hormone‑receptor‑positive and triple‑negative models (e.g., MDA‑MB‑231); future work should extend validation to additional cell lines to confirm the observed effects.

Second, although Annexin V/PI flow cytometry robustly quantifies early apoptotic populations by detecting phosphatidylserine externalization, it does not capture the full complement of apoptotic events. The 17.2% apoptosis observed at 48 h post‑inhibition likely reflects an early stage of programmed cell death,The time-dependent increase in apoptosis (5.1% at 24h to 22.5% at 72h) suggests progressive activation of apoptotic machinery rather than acute cytotoxic effects. Comprehensive pathway validation—such as caspase‑3/7 activity assays and Western blot detection of cleaved PARP—would strengthen mechanistic insight into executioner caspase activation and confirm progression beyond membrane asymmetry. These approaches have been successfully employed in other miRNA inhibition studies and can be integrated.

Third, our interpretation of c‑MYC downregulation is based solely on mRNA measurements at discrete time points. Direct binding confirmation—via luciferase‑reporter assays targeting the c‑MYC 3′ UTR or RNA pull‑down experiments—was not performed but can be make in further studies to distinguish direct from indirect regulatory mechanisms. Notably, indirect regulation by miRNAs through intermediary targets is well documented, yet confirmation in this context requires targeted assays that can be implemented in future work.

Finally, our retrospective, in vitro design precludes assessment of in vivo relevance and long‑term effects on tumor growth dynamics. Prospective animal studies or organoid models—feasible with existing xenograft platforms—would provide critical validation of the therapeutic potential of miR‑32‑5p inhibition and its impact on c‑MYC‑driven tumor biology.

In summary, these limitations highlight the need for broader cell line testing, protein‑level and binding‑confirmation assays, and in vivo validation. Addressing them will not only reinforce our current findings but also refine the mechanistic framework and accelerate translation toward targeted therapies.

#### Technical limitations and future methodological considerations

The targeted assays employed here (qRT-PCR, MTT viability, flow cytometry for apoptosis/necrosis) yield focused insights into miR-32-5p’s phenotypic and transcript-level effects but do not capture the full complement of direct targets or intermediary regulators. The reviewer’s recommendation to apply advanced transcriptomic analyses—such as RNA-sequencing—would address this limitation by enabling:

- **Genome-wide Target Discovery:** RNA-seq of cells treated with LNA-anti-miR-32-5p could identify mRNAs that increase upon inhibition (putative direct targets) versus those that decrease (indicative of downstream, indirect regulation of genes like c-MYC).
- **Pathway and Network Enrichment:** Differential expression profiles, analyzed via gene set enrichment or network analysis, would reveal signaling axes and transcriptional programs modulated by miR-32-5p.
- **Temporal Resolution:** Time-course transcriptomics (e.g., sampling at 6, 24, and 48 h) would distinguish immediate, likely direct effects from delayed, indirect responses.
- **Isoform-Specific and Non-coding RNA Insights:** RNA-seq data could uncover alternative splice variants or non-coding RNAs (lncRNAs, circRNAs) that participate in competing endogenous RNA (ceRNA) networks influencing c-MYC.
- **Integration with Proteomics:** Coupling transcriptomic changes with quantitative proteomics would validate translational consequences and uncover post-translational modifications.

In addition to RNA-seq, future application of CLIP-seq (to map direct miRNA–mRNA interactions), ribosome profiling (to assess translational control), and ChIP-seq (to monitor transcription factor occupancy) would further refine mechanistic understanding. Although such comprehensive approaches extend beyond the present work, our current findings establish a solid foundation—particularly the FBXW7-mediated axis—for subsequent high-resolution studies into the miR-32-5p/c-MYC regulatory network in breast cancer.

## Conclusion

This is the first study to establish miR-32-5p → FBXW7 → c-MYC as a regulatory axis in breast cancer. While miR-32-5p targets *BMP5* in colorectal cancer [37] and *PTEN* in myeloma [41], its role in stabilizing c-MYC via FBXW7 degradation is unique to breast malignancy and offers subtype-specific therapeutic potential.

Our findings suggest miR-32-5p may act as an oncogenic contributor in breast cancer, potentially through c-MYC modulation. Clinical translation of LNA-based miR-32-5p inhibitors may warrant future exploration in c-MYC-overexpressing tumors resistant to conventional therapies. This discovery is notably important due to the documented function of c-MYC as a promoter of oncogenesis in various cancer types [12, 13, 50, 51]. The inhibition of miR-32-5p provoked a considerable decline in MCF-7 cell viability, escalated apoptosis, and a notable reduction of c-MYC expression.

Our findings coincide with prior research in several cancer types, suggesting a conserved oncogenic function for miR-32-5p across diverse malignancies, and present opportunities for prospective therapeutic uses and further investigations, including:

1. Advancement of LNA-anti-miR-32-5p as a therapeutic modality for malignant carcinoma of the breast, leveraging the encouraging outcomes of previous LNA-based oligonucleotides in clinical studies [52, 53].
2. Investigation of miR-32-5p as a prospective biomarker for c-MYC activity in breast cancers, potentially facilitating patient classification and therapy choices.

## Data Availability

No datasets were generated or analysed during the current study.

## Abbreviations

ATCC: American type culture collection
BMP5: Bone morphogenetic protein 5
cDNA: Complementary deoxyribonucleic acid
c-MYC: Cellular myelocytomatosis oncogene
ΔΔCt: Delta-Delta threshold cycle
ELISA: Enzyme-linked immunosorbent assay
FBS: Fetal bovine serum
FITC: Fluorescein isothiocyanate
HEPES: 4-(2-Hydroxyethyl)-1-piperazineethanesulfonic acid
HIF-1α: Hypoxia-inducible factor 1-alpha
LNA: Locked nucleic acid
MCF-7: Michigan cancer foundation-7 (breast cancer cell line)
miRNA/miR: MicroRNA
MTT: 3-(4,5-Dimethylthiazol-2-yl)-2,5-diphenyltetrazolium bromide
PBS: Phosphate-buffered saline
PCD: Programmed cell death
PI: Propidium iodide
PS: Phosphatidylserine
PTEN: Phosphatase and tensin homolog
qRT-PCR: Quantitative real-time polymerase chain reaction
RNA: Ribonucleic acid
RPMI: Roswell park memorial institute (medium)
SEM: Standard error of the mean
TOB1: Transducer of ERBB2, 1
